# STRA-MI-VT (STereotactic RadioAblation by Multimodal Imaging for Ventricular Tachycardia): rationale and design of an Italian experimental prospective study

**DOI:** 10.1007/s10840-020-00855-2

**Published:** 2020-08-27

**Authors:** C. Carbucicchio, B. A. Jereczek-Fossa, D. Andreini, V. Catto, G. Piperno, E. Conte, F. Cattani, E. Rondi, S. Vigorito, C. Piccolo, A. Bonomi, A. Gorini, M. Pepa, S. Mushtaq, G. Fassini, M. Moltrasio, F. Tundo, G. Marvaso, F. Veglia, R. Orecchia, E. Tremoli, C. Tondo

**Affiliations:** 1 Department of Clinical Electrophysiology and Pacing, Centro Cardiologico Monzino, IRCCS, Milan, Italy; 2 Department of Oncology and Hemato-oncology, University of Milan, Milan, Italy; 3 Division of Radiotherapy, IEO European Institute of Oncology, IRCCS, Milan, Italy; 4 Cardiovascular Computed Tomography and Radiology Unit, Centro Cardiologico Monzino, IRCCS, Milan, Italy; 5 Department of Clinical Sciences and Community Health, University of Milan, Milan, Italy; 6 Unit of Medical Physics, IEO European Institute of Oncology, IRCCS, Milan, Italy; 7 University of Milan, Milan, Italy; 8 Biostatistics Unit, Centro Cardiologico Monzino, IRCCS, Milan, Italy; 9 Psycho-Cardiology Service, Centro Cardiologico Monzino, IRCCS, Milan, Italy; 10 Scientific Directorate, IEO European Institute of Oncology, IRCCS, Milan, Italy; 11 Scientific Directorate, Centro Cardiologico Monzino, IRCCS, Milan, Italy

**Keywords:** Ventricular tachycardia, Stereotactic body radiotherapy, Cardiac radiosurgery, Catheter ablation, Radioablation

## Abstract

**Background:**

Ventricular tachycardia (VT) is a life-threatening condition, which usually implies the need of an implantable cardioverter defibrillator in combination with antiarrhythmic drugs and catheter ablation. Stereotactic body radiotherapy (SBRT) represents a common form of therapy in oncology, which has emerged as a well-tolerated and promising alternative option for the treatment of refractory VT in patients with structural heart disease.

**Objective:**

In the STRA-MI-VT trial, we will investigate as primary endpoints safety and efficacy of SBRT for the treatment of recurrent VT in patients not eligible for catheter ablation. Secondary aim will be to evaluate SBRT effects on global mortality, changes in heart function, and in the quality of life during follow-up.

**Methods:**

This is a spontaneous, prospective, experimental (phase Ib/II), open-label study (NCT04066517); 15 patients with structural heart disease and intractable VT will be enrolled within a 2-year period. Advanced multimodal cardiac imaging preceding chest CT-simulation will serve to elaborate the treatment plan on different linear accelerators with target and organs-at-risk definition. SBRT will consist in a single radioablation session of 25 Gy. Follow-up will last up to 12 months.

**Conclusions:**

We test the hypothesis that SBRT reduces the VT burden in a safe and effective way, leading to an improvement in quality of life and survival. If the results will be favorable, radioablation will turn into a potential alternative option for selected patients with an indication to VT ablation, based on the opportunity to treat ventricular arrhythmogenic substrates in a convenient and less-invasive manner.

## Introduction

In patients with structural heart disease, catheter ablation by radiofrequency (RF) energy delivery is effective in the treatment of ventricular tachycardia (VT) recurrences, and is considered of pivotal importance in combination with implantable cardioverter defibrillator (ICD) and antiarrhythmic drugs to prevent sudden cardiac death [1–7]. However, results of RF cannot be extended to all patients with recurrent VTs; the possibility to effectively treat the arrhythmogenic substrate is indeed limited by several factors, including the complexity, extent, and depth of the arrhythmogenic substrate [8–11]. This evidence clearly explains the suboptimal percentage of patients successfully treated for VT. Moreover, the ablation procedure is complex, presents a high degree of invasiveness, and may expose the patient to several life-threatening complications; of note, in patients with severe cardiac dysfunction, and particularly in case of significant respiratory or renal comorbidities, the high procedural risk may even constitute by itself an absolute contraindication for any interventional or surgical approach [12].

The use of stereotactic body radiation therapy (SBRT, radioablation) represents a noninvasive approach, in which the energy delivery technique is not related to any cardiac catheterization [13, 14]. Advanced imaging techniques for the characterization of the substrate and the definition of the anatomical target are employed. This noninvasive approach minimizes the procedural risk associated with the insertion, positioning, and manipulation of the catheters, during both mapping and ablation. In this regard, radioablation allows the delivery of energy to any theoretical target in a three-dimensional system without the need for anatomical contact; furthermore, the prospect of simulating the lesion produced on the diagnostic model provides a patient’s “tailored” therapy, enhancing safety and effectiveness [15].

Of note, different cellular processes are involved in the mechanism of action of radioablation; the therapeutic effect of SBRT is supposed to arise basically from the induction of cellular death and transmural fibrosis that leads to the abolishment of abnormal conduction/automaticity in the myocardial tissue responsible of the arrhythmia [16, 17]. Fibrosis maturation is however progressive and requires several weeks to complete, so that in the intermediate phase, other factors, including acute inflammation and vascular injury, may impact on short-term outcome [16, 18].

Recently, SBRT has been used for the treatment of 35 patients with VT [13, 19–30]. SBRT, guided by imaging techniques partially integrated by electrocardiographic and/or electroanatomical data, significantly reduced in these patients the arrhythmic burden, in the absence of major adverse events correlated with the treatment applied.

Based on these evidences, we designed a spontaneous, prospective, experimental (phase Ib/II), open-label study to validate efficacy and safety of SBRT in a well-defined cohort of patients with recurrent VT not eligible to conventional catheter ablation.

## Methods

**Fig. 1:**
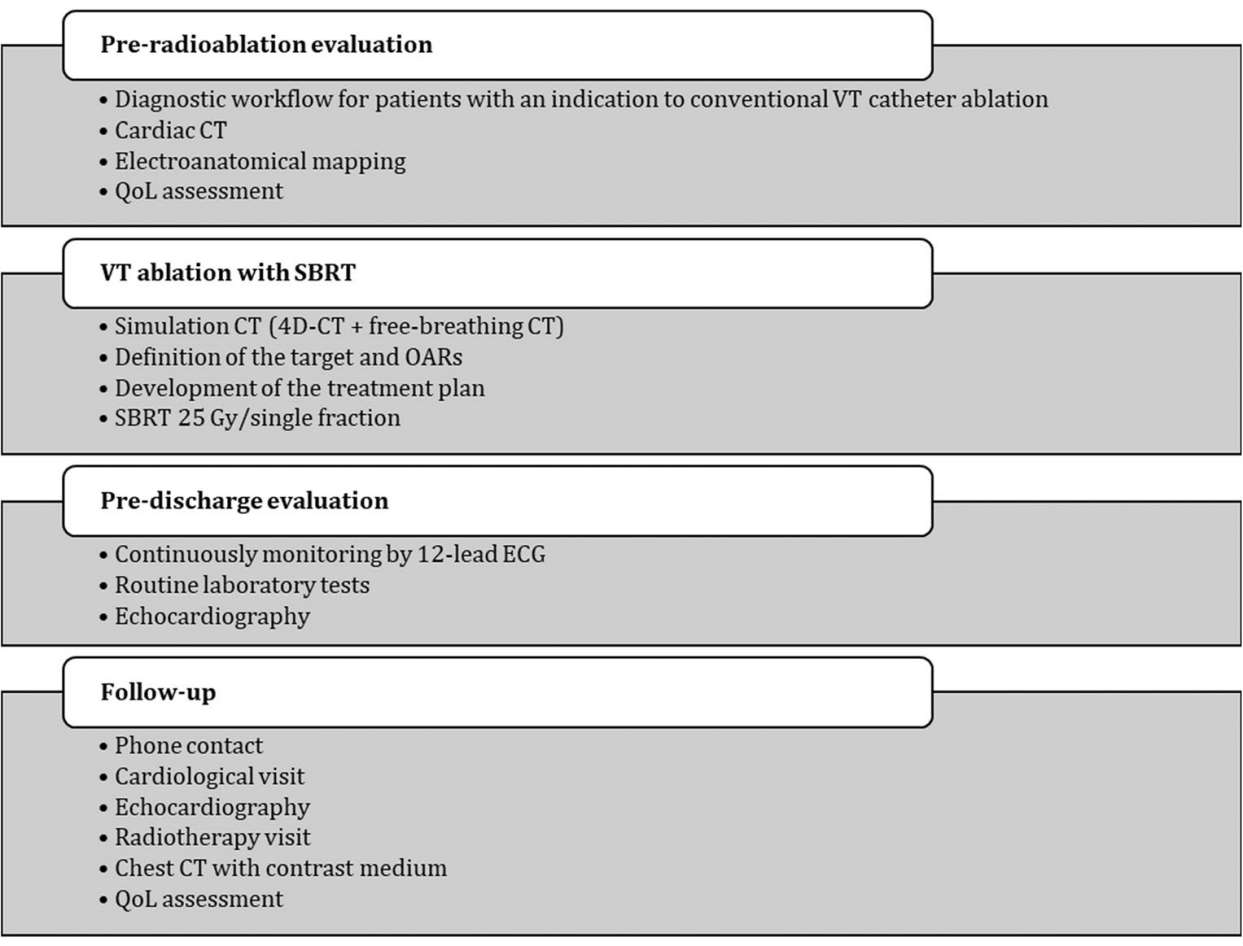
Flowchart of STRA-MI-VT study. Legend: CT, computed tomography; ECG, electrocardiogram; OARs, organs at risk; QoL, quality of life; SBRT stereotactic body radiotherapy; VT ventricular tachycardia

The STRA-MI-VT trial was designed as spontaneous, experimental (phase Ib/II), prospective, open-label study. Fifteen patients are expected to be enrolled. The design of the study is illustrated in Fig. 1 and in Table 1.

**Table 1:** Planning of the study activities

		Study phases							
		Screening and enrolment	Pre-treatment	Radioablation	Post-treatment (until discharge)	1 month FU	3 months FU	6 months FU	12 months FU
Study activities	Compliance with inclusion/exclusion criteria	X							
	Informed consent	X							
	Anamnesis		X						
	Physical examination		X		X				
	Laboratory tests		X		X				
	Thorax RX		X						
	Echocardiogram		X		X		X	X	X
	12-lead ECG		X		X		X	X	X
	ICD interrogation		X	X	X		X	X	X
	Other routine patient-related exams		X		X				
	QoL evaluation		X				X	X	X
	Cardiac CT		X						
	Electroanatomical mapping		X^*^						
	Simulation thorax CT and target and OARs definition		X						
	Treatment planning elaboration		X						
	Radioablation			X					
	Weekly phone contact					X			
	Cardiological visit				X		X	X	X
	Radiotherapy visit						X		X
	Thorax CT						X		X
	Adverse effects evaluation			X	X	X	X	X	X

^*^To be performed unless already in possess of necessary diagnostic information

*CT* computed tomography; *ECG* electrocardiogram; *FU* follow up; *ICD* implantable cardioverter defibrillator; *QoL* quality of life; *RX* radiography

### Study population

The population of the STRA-MI-VT study was defined following the inclusion/exclusion criteria reported below, which present an advantageous cost-benefit ratio in relation to the severity of patients’ arrhythmic condition.

#### Eligibility criteria

The inclusion criteria are as follows:

- Patients with relapsing VT (at least 3 episodes of VT conditioning ICD intervention or VT with incessant or similar-incessant presentation) that has proved to be refractory to any form of pharmacological or non-pharmacological treatment
- Patients with contraindication to conventional ablation, in relation to the high risk associated with the procedure for the type/severity of the condition of heart disease and/or presence of severe comorbidity; or alternatively, patients who have already undergone RF ablation in presence of an arrhythmogenic substrate refractory to previous ablation procedures or not suitable for any interventional approach,^1^ refusing any surgical ablative attempt because of a proven high operative risk, in relation to the patient’s characteristics
- Ejection fraction of the left ventricle ≥ 20%
- Age ≥ 50 years
- ICD/subcutaneous ICD recipients
- Signed informed consent

The exclusion criteria are as follows:

- Inability to express informed consent to participate in the study
- Previous thoracic radiotherapy with cardiac involvement
- Active myocardial ischemia
- Cardiac revascularization < 120 days.
- Non-correctable hemodynamic instability (cardiogenic shock, New York Heart Association (NYHA) IV)
- Comorbidity with prognosis < 12 months or other contraindications deemed significant
- Pregnant women

### Pre-treatment evaluation

Patients will preliminarily undergo the complete diagnostic workup defined for patients with an indication to conventional VT catheter ablation. In agreement with our ordinary practice, electroanatomical mapping (EAM) combined with cardiac computed tomography (CT) will represent the first choice of imaging to identify the arrhythmia substrate and to define the target area.

The patients’ quality of life will be evaluated using the Short Form-36 (SF-36) health questionnaire.

#### Cardiac computed tomography

**Fig. 2:**
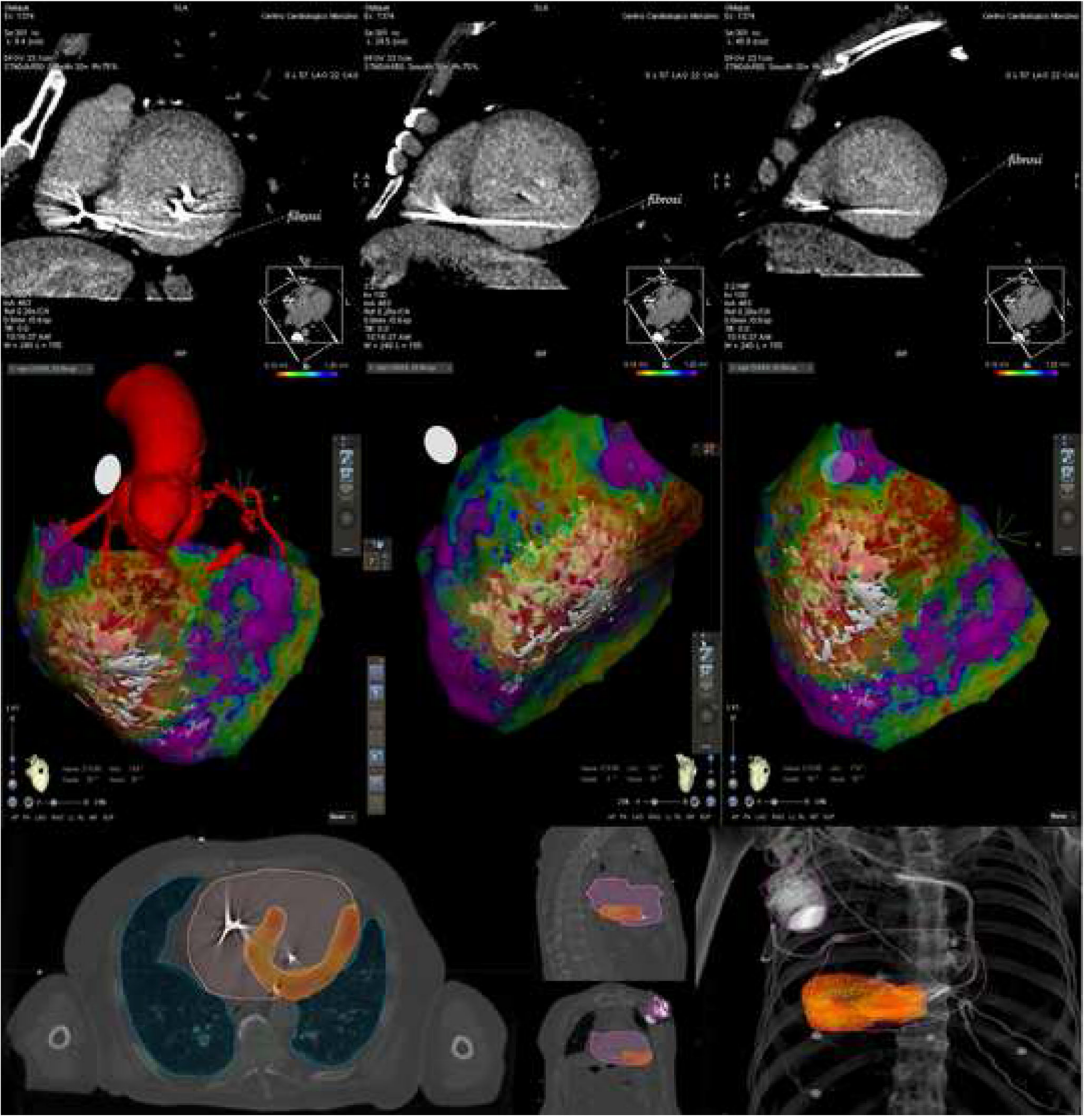
Diagnostic imaging obtained by cardiac CT combined with EAM to guide patient’s SBRT treatment. **Upper panel**: LV short axis view of cardiac CT scan obtained 8 min after iodinated contrast administration. Hyper-density on inferior and postero-lateral LV wall is well evident and represents transmural myocardial fibrosis with ischemic pattern extending from the base to the apex of LV. **Central panel**: High-density epicardial EAM combined with CT imaging. A large portion of the inferior wall is covered by diseased electrograms, with decreased amplitude (<< 1.0 mV, red-to-yellow in the “color-coded” map), as expression of an underlying electrical “dense scar.” In the same location, CT shows a pattern of discrete transmural fibrosis, thus perfectly matching the lesion revealed by EAM. **Lower panel**: The inferior panel shows the coverage (95% isodose) of the target volume obtained after optimization of treatment plan. The target volume is the result of imaging integration between simulation and diagnostic CT, and EAM. Legend: CT, computed tomography; EAM, electroanatomical mapping; LV left ventricle; SBRT stereotactic body radiotherapy

Cardiac CT will be performed using a whole heart coverage (16 cm along Z-axis) CT scan (Revolution CT, GE Healthcare, Milwaukee, WI, USA) with the following parameters: slice configuration 256 × 0.625 mm, gantry rotation time 280 m s, and prospective electrocardiography (ECG) triggering. A new generation of iterative reconstruction, called ASIR-V (GE Healthcare), will be used for image reconstruction. Pre-scan sublingual nitrates and betablocker (i.v. metoprolol up to 20 mg) will be administered, if not contraindicated. Patients will receive a 1.5-mL/kg bolus of contrast medium (Iomeron 400 mg/mL, Bracco), divided in two separate bolus 5 mL/s followed by saline infusion, both at an infusion rate of 5 mL/s. A first CT scan will be obtained at the angiographic phase to have adequate coronary artery contrast opacification as routinely performed for coronary CT angiography. A second series of breath-hold and ECG-gated images will be acquired after 8 min from contrast agent injection (100 Kvp; 500 mA) for the detection of myocardial delayed enhancement (DE, Fig. 2, upper panel). Visual evaluation of DE will be performed and a narrow window width and level (350 W and 150 L) will be used for late scan evaluation that is best viewed as thick average intensity projections (0.5–0.8 cm). The presence of DE will be then confirmed as hyperdense myocardium with signal intensity > 2 standard deviation (SD) above remote myocardium, as previously described [31–33]. Myocardial wall thinning (wall thickness < 5 mm) and DE involving > 50% of myocardial thickness will be considered transmural DE. A dedicated post-processing reconstruction will be applied to extrapolate single DICOM files including only myocardial fibrosis volume, coronary and ascending aorta anatomy, pericardial fat volume, and selected extracardiac anatomical structure (i.e., spine and sternum).

#### Electroanatomical mapping

A left ventricular (LV) endocardial electroanatomical mapping (EAM), which allows to reconstruct three-dimensional cardiac chambers and to detect intracardiac electrocardiogram, will be acquired with a conventional percutaneous approach. The standard for identifying major sites of arrhythmogenicity is represented by high-density EAM, acquired with the CARTO system (Biosense Webster), which provides essential information of voltage abnormalities, representing diseased tissue, and local activation times; as a rule, the LV map was consequently obtained by using Pentaray or Decanav catheter (Biosense Webster), acquiring a total of ≥ 500 points with more than 5 points/cm^2^ density in the areas of interest. Basically, the reference value of bipolar endocardial voltage to define diseased LV was 1.5 mV, being “dense scar” characterized by intracardiac electrogram (EGM) signals < 0.5 mV. With regard to the possibility to reveal any intramural/epicardial lesion, signals were considered pathological if signal amplitude was < 8.0 mV and/or < 1.0 mV for unipolar endocardial and bipolar epicardial electrograms, respectively.

Pre-acquired DICOM files will be then merged (imaging fusion) with EAM to characterize the VT substrate and validate the correlation between diseased myocardium diagnosed by EAM and fibrotic lesions revealed by CT (Fig. 2, central panel). This combined anatomical and functional characterization of the arrhythmia substrate will be used to identify areas of diseased myocardium responsible for the arrhythmias and will represent the target for SBRT. The evaluation will be performed by a panel of two electrophysiologists, one clinical cardiologist, one radiologist, and two radiotherapists, with more than 10 years of experience in their specific field, achieving a consensus on the target area.

Previous mapping and imaging integration data will be used for target identification only if acquired at the end of the last patient’s procedure, in order to ensure accuracy of substrate characterization. Eventually, it will be admitted to renounce EAM in selected patients with a contraindication to any interventional procedure in whom SBRT will be guided by imaging techniques only.

### Treatment

Patients will undergo a simulation CT (chest CT), acquired in order to include the whole lung fields (from the apex to the base), aimed at simulating the patient’s anatomy and allowing the correct positioning of the patient during the radioablation procedure. Subsequently, a series of CT scans will be acquired which include a free breathing CT and a “related breathing” CT (4D-CT) which provide information on respiratory movement. The information obtained by simulation CT, EAM, cardiac CT, and any other available imaging will be used to identify and draw: (1) both on free breathing CT and on 4D-CT, the ablation site represented by the clinical target volume (CTV) and (2) on the free breathing CT all surrounding anatomical structures, also known as organs at risk (OAR), including the ICD. The CTVs drawn on all the images of different respiratory phases will be transported on free breathing CT, and an internal target volume (ITV) will be defined as the union of the CTVs, taking into account the target respiratory motion. Starting from ITV, a planning target volume (PTV) will be built, expanding the ITV in three dimensions. This margin takes into account any residual uncertainty related to patient positioning, unwanted movements, and dose emission (Fig. 2, lower panel) [34]. The most appropriate physical-dosimetric study for the patient’s anatomy will be processed using treatment plan system (TPS) and will be generated in order to deliver a total dose of 25 Gy in a single fraction [14, 35], to achieve maximum coverage of the PTV region by minimizing the dose to the surrounding healthy tissues.

SBRT will be delivered using one of the linear accelerators installed at the IEO, i.e., 3 tomotherapy units, 1 Cyberknife, 1 Trilogy, and 1 Vero. All linear accelerators except Cyberknife will use a modulated intensity radiotherapy (IMRT) isocentric-based technique. In contrast, Cyberknife will deliver the treatment via non-isocentric beams, originated at arbitrary positions in the workspace and focused at arbitrary points in the target.

The choice of equipment to be used for each patient enrolled in the STRA-MI-VT study will be made taking into consideration different elements, such as size of the lesion to be treated, location of the lesion, patient anatomy, OARs near the lesion, presence of markers close to the lesion visible from the imaging systems, and concomitant pathologies [36]. The plan will be prepared simulating patient’s treatment on different linear accelerators to optimize the equipment for each specific case. All the linear accelerators are provided with image-guided radiotherapy (IGRT). The acquired images will be volumetric, i.e., cone-beam CT (CBCT) and/or fluoroscopic (kV), checking for both modes the accuracy of patient positioning before the delivery of the treatment dose.

### Pre-discharge evaluation

After SBRT, all patients will be continuously monitored by 12-lead ECG for a period of at least 5 days; routine laboratory tests and echocardiography examination will be provided within 24 h after SBRT and before hospital discharge. Particular care will be addressed to exclude secondary effects related to SBRT.

ICD interrogation will be performed immediately after SBRT and before patient discharge to verify device and lead parameters and exclude malfunctions. As a rule, ICD ventricular arrhythmias detection and therapies will be maintained the same as before SBRT. Furthermore, ICD remote monitoring will be set up for all patients.

### Follow-up

Adverse effects will be classified according to the international Common Terminology Criteria for Adverse Events v 5.0/CTCAE document.

A follow-up phone-call will be performed once a week for the first 4 weeks after SBRT.

Regular cardiological follow-up visits, including 12-lead ECG and ICD interrogation, will be performed 3, 6, and 12 months after SBRT. Echocardiography examination and SF-36 health questionnaire will be provided 3, 6, and 12 months after SBRT. Patients will be visited by a radiation oncologist, and thorax CT will be performed 3 and 12 months after SBRT.

Pharmacological therapy, including antiarrhythmic drugs, will be maintained the same as before SBRT during the entire follow-up.

With regard to the early post-SBRT phase and the possible occurrence of transient processes impacting on cardiac arrhythmogenicity, accordingly to literature [16–18, 21], a 6-week “blanking period,” starting immediately after SBRT treatment, will be considered in the analysis of VT recurrences during follow-up.

### Endpoints of the study

#### Primary endpoints

Two primary endpoints of the study have been defined, represented by safety and efficacy of the treatment, respectively.

- Primary safety endpoint is to evaluate the safety of radiation therapy in the acute phase (during the first 30 days of the procedure) and at 3, 6, and 12 months. The incidence of serious adverse events related, or presumably related, to radioablation will be assessed as a percentage of the enrolled patients. Serious adverse events were the following:
  - Death
  - Cardio-circulatory arrest
  - Acute myocardial infarction
  - Cardiogenic shock
  - Pericarditis, myocarditis, and/or endocarditis
  - Acute heart failure
  - Gastro-esophageal lesion (ulcer/stenosis/fistula/hemorrhage/perforation)
  - Bronchopulmonary infection
  - Injury to the respiratory system (fistula/bronchial or tracheal stenosis, pulmonary fibrosis)
  - Radio-induced oncogenesis
- Primary efficacy endpoint is to evaluate the procedural effectiveness of therapy at 3, 6, and 12 months, represented by the total number of VT/VF episodes occurring after “blanking period,” compared with the period preceding the radioablation procedure (6 months).

VT episodes will be categorized as follows:

- VT episode > 30 s, regardless of the intervention of the ICD
- VT episode conditioning ICD intervention by means of “anti-tachycardia pacing” electrical therapy (without shock delivery)
- VT episode conditioning ICD shock intervention

The 6-week “blanking period” will start on the procedure day.

As a rule, pharmacological therapy, including antiarrhythmic drugs, will be maintained the same as before SBRT during the follow-up period. Changes of the pharmacological therapy will be determined only by specific clinical reasons.

#### Secondary endpoint

Secondary endpoints are the following:

- Global mortality during follow-up (up to 12 months)
- Variations in the quality of life of patients in the follow-up (up to 12 months) with respect to the basal condition
- Changes in cardiac function assessed by estimating the ejection fraction of the left ventricle on the echocardiographic examination in the follow-up (up to 12 months)

### Statistical planning

#### Stopping criteria for ending the study

A study stopping rule based on the global rate of serious adverse events occurring within 30 days from the date of SBRT, that may be a consequence of the radioablation treatment (death, acute myocardial infarction, cardiogenic shock, cardio-circulatory arrest), will be adopted after enrollment of 5 patients. This rule will require the interruption of enrollment in the study if the observed adverse event rate will be higher than expected based on the data reported in the literature (at the time of study design) [12, 17–20]. Specifically, the reported rate (0 events out of 12 patients) is compatible with an estimated maximum frequency of 1 out of 5 events (upper limit of the 95% confidence interval). Therefore, the study will be stopped if ≥ 2 events occur within 30 days of the treatment carried out, among the first 5 patients treated.

#### Calculation of the target sample size and statistical analysis

A sample of 15 patients will allow a 60% reduction in ventricular arrhythmic episodes to be assessed as significant (*p* < 0.05), assuming an average of 10 episodes in the 3 months preceding the procedure, with a statistical power of 90%. Continuous variables will be expressed as mean ± standard deviation or median and interquartile range variables with non-normal distribution, and discrete variables as absolute numbers and percentages. Primary efficacy endpoint (reduction of ventricular arrhythmic episodes) and secondary endpoints (clinical, echocardiographic, and psychometric characteristics) will be evaluated with the Student’s *t* test for paired data (continuous variables with normal distribution) or with the Wilcoxon sign rank test (variables with non-normal distribution). Kaplan-Meyer curves will be developed for event-free survival analysis at follow-up. All tests will be two-tailed and a *p* < 0.05 will be considered significant.

### Study organization and status

Patients are selected from a population submitted to the Centro Cardiologico Monzino (CCM) as nationwide referral center for VT ablation; the SBRT treatment will be carried out at the Istituto Europeo di Oncologia (IEO) that is one of the largest radiotherapy facilities in Italy.

Recruitment started in September 2019 and is expected to end in September 2021. Two years are planned for enrollment as all participants will be selected on the basis of strict inclusion/exclusion criteria being nonresponders to, or not suitable for, any conventional approach. At the date of submission of the manuscript, 4 patients underwent SBRT and 3 completed a 3-month follow-up.

Preliminary results are expected within 1 year from the study beginning; final data analysis will be provided once follow-up period will be completed for all patients (approximately September 2022). On August 26, 2019, the STRA-MI-VT trial was prospectively registered at clinicaltrials.gov (NCT04066517).

## Discussion

STRA-MI-VT is the first Italian spontaneous, experimental, prospective, open-label study testing the role of SBRT for the treatment of recurrent VT refractory to conventional therapies, in patients with structural heart disease.

In oncology, SBRT represents a well-established treatment modality that allows to noninvasively destroy cancerous cells by delivering multiple radiation beams. In the treatment of ventricular arrhythmias, radiation therapy is supposed to modify the arrhythmogenic substrate, by eliminating the heterogeneity responsible of the reentry mechanism, similarly to RF, thus reducing the episodes of VT. Differently from RF, SBRT allows to potentially ablate deep structures, which cannot be reached by catheters [29] and to accomplish the treatment in a noninvasive, faster, and safer manner [14].

On the other side, differently from standard ablation procedures, which lead to an immediate reduction of VT events, SBRT is supposed to manifest its therapeutic effects after months [29, 37]. Indeed, in the 6–8 week period following SBRT, corresponding to the “blanking period,” VT episodes may theoretically even be facilitated, given the complex maturation process of the lesion caused by SBRT [16–18, 21]. Moreover, the precise effect of high dose of radiation for the treatment of a structurally diseased heart is not well known; an additional concern is related to the possible wrong localization of target due to respiratory and cardiac motion: this could lead to target tissue underdosing and normal tissue overdosing, undermining both efficacy and safety of the treatment.

### Analysis of previous experiences

To the best of our knowledge, nine clinical studies are currently underway regarding the use of stereotactic radiation therapy for the treatment of ventricular arrhythmias, registered at clinicaltrials.gov as NCT02919618, NCT02661048, NCT03601832, NCT03819504, NCT03349892 (suspended), NCT04066517, NCT04162171, NCT04065802, and NCT03867747. Results are available and published exclusively for the first two of them.

Some studies in literature already showed that radioablation allows to reduce the occurrence of arrhythmic episodes without significant side effects directly imputable to the treatment and hence demonstrated both its efficacy and safety. Loo and colleagues [20] carried out the first-in-human treatment of ventricular arrhythmia. No acute and late complications were reported, but the efficacy of the treatment was limited to the first months of the treatment. Efficacy and short-term safety were also confirmed in other studies in which only one patient was treated [22, 23, 26–28, 30]. Neuwirth and colleagues [24] reported the second largest case series on the safety and efficacy of SBRT to treat patients with recurrent VT. During 3 months after SBRT, VT burden was reduced by 87.5% and the treatment was well-tolerated; out of the 10 treated patients, only 4 reported nauseas and only 1 presented gradual progression of mitral valve regurgitation. Severe short-term complications were instead observed by Robinson and colleagues [13], in the context of the abovementioned ENCORE-VT trial. As a matter of fact, out of the 19 enrolled patients, VT episodes were reduced in almost all patients with consequent improvement in quality of life, but 2 patients experienced transient heart failure exacerbation and pericarditis, most likely caused by SBRT. Last but not least, in a series of 5 high-risk patients with scar-related VT, the safety profile of SBRT was confirmed to be favorable by Gianni and colleagues [38]; despite good initial results, all patients experienced however clinically significant recurrences over the mid- to late-term. Given these in part contradictory evidences about long-term efficacy and safety, and due to the fact that SBRT has been mainly used as a palliative therapy in patients with short life expectancy, further studies in the field are eventually required.

In nearly all the investigations, prescription dose was 25 Gy delivered in a single fraction, even though preclinical studies demonstrated the safety of more escalated treatments [12]. Scholz and colleagues [28] prescribed a single dose of 24 Gy to the PTV-encompassing 80% isodose. Zeng and colleagues [29] opted for an even more cautious approach (24 Gy in 3 fractions), justified by the fact that VT was secondary to a cardiac tumor and that the tissue to ablate was in a close anatomical relationship to the left anterior descending artery. Actually, one major issue is to evaluate the optimal dose to deliver to the heart, which should be effective but safe at the same time, minimizing induced toxicity to surrounding tissues and possible damages to the ICD [39, 40]. Data available from literature are not enough to define the best dosimetric variables as, in oncological applications, the heart is rarely the target organ, being intracardiac malignancies quite uncommon. On the contrary, the heart often represents one of the OARs with the highest avoidance priority when irradiating thoracic tumors. Cardiac and particularly respiratory-induced thoracic motion represent an additional level of complexity, as the treatment system must be able to accurately track the moving target [37]. According to this protocol, this problem is overcome by defining a cardiac and respiratory ITV, which considers both causes of motion [37].

### Possible effects of SBRT: Addressing “pros” and “cons”

The benefit supposed to derive from the use of SBRT is represented by the superior efficacy in the control of VT with respect to the conventional percutaneous procedure. This superior efficacy specifically derives from the hypothesis that the SBRT can result in a more appropriate and discrete substrate modification in patients in whom the conventional approach has failed. In a clinical setting, SBRT will be offered as an option in patients who are not eligible to any interventional or surgical approach due to an unacceptably high procedural risk.

Theoretical disadvantages associated with SBRT relate to the fact that we will use a novel therapeutic approach that has been tested only in selected groups of patients; therefore, there is no evidence of its efficacy as a standard treatment. Of note, for STRA-MI-VT patients, this limitation has little significance as all participants will be first categorized as nonresponders or not suitable for any conventional approach. In addition, SBRT may be responsible for cardiac and noncardiac adverse effects, due to the higher radiation exposure of the patients. Even if these effects are, based on the literature, transient, and of mild to moderate entity, specific attention will be given in our study to prospectively evaluate cost-effectiveness of SBRT.

## Conclusions

The existing research confirms that SBRT might represent a relatively effective and well-tolerated therapeutic option in patients with VT who have failed other approaches [37].

STRA-MI-VT study aims at demonstrating that SBRT may represent an alternative therapeutic option in selected patients with substrate-related VT, thanks to a potentially favorable cost-effectiveness ratio. An advanced combined multimodal imaging will be used to characterize the arrhythmogenic substrate and to identify the target for SBRT, thus STRA-MI-VT is supposed to provide significant information to define the adequacy of this novel imaging-integration methodology.

SBRT is widely used in oncology to treat small primary or secondary malignant lesions to high doses in few fractions, leading to local control of about 90% [41]. In the last years, it has been proposed to some benign conditions like trigeminal neuropathy, acoustic neurinomas, or arteriovenous malformations [42]. VT is a condition that might prospectively benefit from this noninvasive approach.

Preliminary results are expected to be available within 1 year. If primary endpoints of STRA-MI-VT will be reached, SBRT has the potential to be offered as a novel therapeutic option to a larger population of patients with mostly intractable VTs, based on the attractive possibility to treat also substrates inaccessible to conventional approaches in a safe and less-invasive manner.

Finally, the methodology of the STRA-MI-VT is based on the cooperation of different teams, with specific competences in arrhythmology, cardiac imaging, and radiation therapy, in an attempt to validate and share a multidisciplinary workflow process that may represent the premise for future clinical experiences in the field.

## Data Availability

Not appropriate.

## Footnotes

1 For example patients with deep intramural substrate, with epicardial fat precluding an effective epicardial ablation, with pericardial adhesions limiting pericardial access, with mitroaortic mechanical prostheses.

## Code availability

Not appropriate.
